# Effect of different drying temperatures on the rehydration of the fruiting bodies of Yu Muer (*Auricularia cornea*) and screening of browning inhibitors

**DOI:** 10.1002/fsn3.1891

**Published:** 2020-09-27

**Authors:** Yanqi Chen, Zhiwen Lv, Zhirun Liu, Xiao Li, Changtian Li, Frederick Leo Sossah, Bing Song, Yu Li

**Affiliations:** ^1^ Engineering Research Center of Edible and Medicinal Fungi, Ministry of Education Jilin Agricultural University Changchun China; ^2^ Guizhou Key Laboratory of Edible fungi breeding Guizhou Academy of Agricultural Sciences Guiyang China; ^3^ College of Plant Protection Jilin Agricultural University Changchun China

**Keywords:** *Auricularia cornea* cv. Yu Muer, browning inhibitor, hot‐air drying, polyphenol oxidase, rehydration methods

## Abstract

In this study, the color of the dry fruiting bodies, fresh weight (FW): dry weight (DW) ratio, amino acids, and total phenolics, which are of nutritional or commercial interest, were compared among different drying temperature treatments. The effect of rehydration methods and color protection reagents on the fruiting body color, polyphenol oxidase (PPO) activity, and browning inhibition rate were evaluated. The results showed that drying with hot air at 65℃ was quickest and resulted in a better color without compromising the FW:DW ratio and rehydration ratio of the fruiting bodies. Furthermore, some reactions that occurred under high temperatures increased the content of protein, amino acids, and total phenolics. Soaking after boiling was the most suitable rehydration method, leading to the lowest PPO activity (39.87 ± 1.35 U/g). All of the four analyzed color protection reagents could significantly inhibit the browning of Yu Muer fruiting bodies under room temperature water rehydration conditions, with a citric acid content of 6 g/L showing the best performance. These results provide technical support for the development of the Yu Muer industry and for promoting the commercial processing of Yu Muer fruiting bodies slices.

## Graphical abstract

The drying with hot air at 65℃ was quickest and resulted in a better color without compromising the drying ratio and rehydration ratio of the fruiting bodies. Soaking after boiling was the most suitable rehydration method, leading to the lowest PPO activity (39.87 ± 1.35 U/g). All of the four analyzed color protection reagents could significantly inhibit the browning of Yu Muer fruiting bodies under room temperature water rehydration conditions, with a citric acid content of 6 g/L showing the best performance.

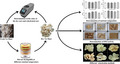

## 1 INTRODUCTION

Yu Muer (*Auricularia cornea* cv. Yu Muer) is a new white variety of edible fungus that was selected from a mutant of *Auricularia cornea* by the Engineering Research Center of the Ministry of Education, Jilin Agricultural University. Yu Muer is in genus *Auricularia*, family Auriculariaceae, order Auriculariales, class Agaricomycetes, and phylum Basidiomycota. It is an edible fungus and is also used in medicine (Royse, 2014; Wang, Jiang, et al., 2019a; Wang, Li, et al., 2019b). The fruiting bodies of Yu Muer are thick, tender, and crispy tastes like jellyfish, and have a jade‐like warm, soft color. It is rich in nutrients, including physiologically active substances such as polysaccharides, proteins, amino acids, di(2‐ethylhexyl) adipate, and myristicin aldehyde. Pharmacological tests have indicated that it possesses high anti‐inflammatory and anticancer activities, can effectively lower blood sugar, and has certain therapeutic effects on antidiabetic and alcoholic liver disease (Wang, Jiang, et al., 2019a; Wang, Li, et al., 2019b; Wang et al., 2018). The commercial production of Yu Muer has reached over 50 million bags thus far in multiple provinces in China, with the products being sold to South Korea and Japan. In some rural areas, the production of Yu Muer has been a substantial economic driver (Fang et al., 2018).

Fresh edible fungi have a short storage time, and shelf lives due to their high water content and active respiration (Reis, Barros, Martins, & Ferreira, 2012). In most cases, they need to be dried in preparation for long‐term storage and marketing. Hot‐air drying (HD) is a typical drying method that is widely used for processing cultivated edible fungi such as *Pleurotus eryngii*, shiitake mushroom (*Lentinula edodes*), oyster mushroom (*Pleurotus ostreatus*), and *Agaricus bisporus*, as well as wild mushrooms such as *Macrolepiota procera* (Fernandes et al., 2013). In addition, the HD method is conducive to the retention of nutrients and the formation of flavor substances (Li et al., 2015; Tolera & Abera, 2017). Sun drying (SD) has been adopted for the drying of most edible fungi (Royse, 2014), but the process requires 2–5 days and is easily affected by the weather and environmental conditions of the drying site. *Auricularia polytricha* is the fourth‐most important edible fungus in the world (Royse, 2014). As its cultivation scale has increased, larger areas of land are required for outdoor SD, and the product also requires longer drying times and higher labor costs, which limit the development of the industry. There is, therefore, an urgent need to develop an efficient and inexpensive method of drying.

The browning of Yu Muer fruiting bodies during rehydration in the water at room temperature is mainly caused by polyphenol oxidase (PPO). PPO is the key enzyme causing browning in agricultural products (Ludikhuyze, Loey, Indrawati, Smou, & Hendrickx, 2003). PPO is present in apples and potatoes as well as in mushrooms, such as *Agaricus bisporus* (Cheung & Henderson, 1972; Ding et al., 2016; Février, Le Quéré, Le Bail, & Guyot, 2017), shiitake mushroom (Ye et al., 2012), wood ears, golden mushroom (*Flammulina velutipes*), and honey fungus (*Armillaria mellea*) (Colak, Sahin, Yildirim & Sesli, 2007), and may lead to the browning or even rotting of fresh edible fungi, thus downgrading their quality.

Color protection reagents have been used to inhibit browning during the processing of fruits and vegetables. Popular browning inhibitors include ascorbic acid (vitamin C), citric acid, sodium ascorbate, and L‐cysteine. Ascorbic acid exists widely in fresh fruits and vegetables and is a Generally Regarded As Safe (GRAS) substance that can be utilized in the preservation of *A. bisporus* (Ojeda, Sgroppo, Martín‐Belloso, & Soliva‐Fortuny, 2019). Citric acid, the most commonly used additive with sour flavoring, is also an antioxidant that can maintain the quality of food and agricultural products. If a certain concentration is reached, citric acid can inhibit the PPO activity in *A. bisporus* (Liu et al., 2013). Sodium ascorbate is mainly used as a preservative and antioxidant for fruits, vegetables, canned foods, and grape wine (Carocho, Morales, & Ferreira, 2017). L‐cysteine is a recognized safe amino acid that can be used as a color protection reagent and preservative for vegetables and fresh‐cut fruits (Ali, Khan, & Malik, 2016). Developing appropriate rehydration methods and screening chemical agents for protecting color during rehydration in the water at room temperature is essential in the development of the Yu Muer industry.

In our study, fresh Yu Muer was processed by HD. Their change in commercial properties, such as color and wet‐to‐dry ratio, and their nutritional composition, such as amino acids, total phenolics, and proteins, were compared under different drying temperatures, and suitable drying temperature for the processing of Yu Muer fruiting bodies was screened. Furthermore, the combinations of Yu Muer fruiting bodies rehydration methods and color protection agents were tested and optimized. Our study provides a theoretical basis and practical reference for improving the drying process, marketability of the fresh products, cold chain transportation, and shelf life of Yu Muer. It may also provide a new technical approach for the rehydration processing of dried Yu Muer fruiting bodies.

## 2 MATERIALS AND METHODS

### 2.1 Materials and chemical reagents

Fresh Yu Muer fruiting bodies were collected from the Fungus and Vegetable Base, Jilin Agricultural University (Changchun City, Jilin Province, China). Test kits for determining PPO, amino acid (AA) content, plant total phenolics, protein content (BCA assay), and total sugar content were purchased from Suzhou Keming Biotechnology Co., Ltd. Sodium erythorbate and L‐cysteine were purchased from Beijing Solarbio Science and Technology Co., Ltd. Ascorbic acid was purchased from Shanghai Yuanye Biotechnology Co., Ltd. Citric acid was purchased from Tianjin Fuchen Chemical Reagent Factory.

### 2.2 Instrumentation

The following instruments were used in the study: Furuite 770C Fruit and Vegetable Dryer (Dianguo Electric Technology Co., Ltd., Foshan, China), Spectramax i3x Multi‐Function Microplate Reader (Molecular Devices, USA), Legend Micro21R Benchtop Microcentrifuge (Thermo Fisher Scientific, USA), Baijie‐100 high‐speed multifunction pulverizer (Deqing Baijie Electric Co., Ltd., Huzhou, China), and ColorFlex 45/0 spectrophotometer (Hunter Lab, USA).

### 2.3 Yu Muer fungus cultivation and collection methods

Indoor cultivation was used to produce the Yu Muer fungus. The spawn running period was conducted at a temperature of 24–26℃ and relative air humidity of 30%–40%. During collection, the temperature was maintained at 20–25℃, and the humidity was at 85%–90%.Furthermore, CO_2_ levels were maintained at less than 1,200 ppm. Mature fruiting bodies of 4–5 cm, thickness of fruiting bodies is 0.1–0.2 cm and with white spores on the ventral surface were collected.

### 2.4 Yu Muer fruiting bodies drying and rehydration methods

SD: Fresh fruiting bodies were evenly spread in a single layer on a drying net. The temperature was 20–35℃. The fruiting bodies were naturally dried until a constant weight was reached.

HD at constant temperature: The fresh fruiting bodies were spread in a single layer (2 kg/m^2^) on the trays. They were respectively dried at 35, 45, 55, and 65 ℃ in the dryer until they reached a constant weight.

The dried fruiting bodies were sampled for rehydration, and the weight of each sample was 10 g. They were treated by 1) soaking in room temperature water for 5 hr, 2) soaking in boiling water for 5 hr, or 3) boiling in water for 10 min and then soaking for 5 hr.

Calculation of water loss ratio: The weight of the fruiting bodies under different drying temperatures was recorded every 0.5 hr. The water loss ratio was calculated by the following equation according to the published method (Xu, Jin, Zhang, & Chen, 2017).

(1) $$Water\;\text{loss ratio}\text{\textbackslash{}\%}\;=\frac{\text{Wa}\;‐\;\text{Wo}}{\text{Wc}}\times 100$$

Wa denotes the current weight; Wo denotes the weight at 0.5 hr before weighing the current Wa; and Wc denotes the weight of the fresh fruiting bodies.

The FW:DW ratio was calculated by the following equation. The fruiting bodies were dried to a constant weight, and the FW:DW ratio was calculated according to the following formula:

(2) $$\text{FW}:\text{WD}\;\text{ratio}\;=\;\frac{\text{Wc}}{\text{Wf}}$$

Wc represents the weight of the fresh fruiting bodies, and Wf represents the weight of the dry fruiting bodies.

The rehydration ratio was calculated by the following method reported by Doymaz (2010). After rehydration, the fruiting bodies were drained for 20 min and then weighed. The rehydration ratio was calculated using the following formula:

(3) $$\text{Re}hydration\;\text{ratio}=\frac{\text{We}}{\text{Wf}}$$

*We* denotes the weight after rehydration, and Wf denotes the weight of the dry fruiting bodies.

### 2.5 Determination of the color of the dry fruiting bodies and rehydrated fruiting bodies

The dry fruiting bodies processed by SD and HD at four different temperatures were pulverized for 1.5 min in a pulverizer, following which the powder was collected for measurements. Before measurement, the color spectrophotometer was calibrated. The collected dry fruiting bodies powder was spread evenly on the tray. Fresh fruiting bodies and rehydrated fruiting bodies that were treated by HD at four different temperatures were selected and spread onto the tray. The water on the surface of the fruiting bodies was removed using water‐absorbent papers, and then, the fruiting bodies were placed flat on the tray for measurement.

The D65 illuminant and 10° viewing angle were used. L* (brightness), a* (positive value refers to redness, and negative value refers to greenness), and b* (positive value refers to yellowness, and negative value refers to blueness) values of the samples were measured. The calculation of ΔE followed the method reported by Ref.(Mohebbi, Ansarifar, Hasanpour, & Amiryousefi, 2011).

(4) $$\Delta E=\sqrt{{\left(L_{0}^{*}‐L^{*}\right)}^{2}+{\left(a_{0}^{*}‐a^{*}\right)}^{2}+{\left(b_{0}^{*}‐b^{*}\right)}^{2}}$$

### 2.6 Determination of PPO activity and nutrient composition in the Yu Muer fruiting bodies

PPO catalyzes the production of a quinone from catechol. Quinone has a characteristic absorption peak at 525 nm, which can be utilized to determine the activity of PPO. The fruiting bodies subjected to different rehydration processes were used in the determination of PPO activity. The water on the surface of the rehydrated fruiting bodies was removed using absorbent papers, and the determination was carried out according to the instruction of the PPO activity assay kit.

The absorbance at 562 nm was measured using the BCA method to determine the protein content (Kim, Abdelhamid, & Pack, 2019). Amino acid content was determined using the ninhydrin colorimetric method, and absorbance at 570 nm was measured (Zhang, Fu, Zhou, Kang, & Li, 2013). Under alkaline conditions, phenolic substances reduce the tungsten molybdic acid to produce a blue compound, and the absorbance at 760 nm is measured to determine the content of total phenolics (Fabrowska, Messyasz, Pankiewicz, Wilinska, & Łęska, 2018). After heating together with reducing sugar, the DNS reagent is reduced and forms an amino compound, which exhibits an orange or red color in an alkaline solution with excessive NaOH. The absorbance at 540 nm is then measured to determine the total sugar content (Yadav, Singh, Balan, Pareek, & Vivekanand, 2019). The powder of the dry fruiting bodies subjected to HD at different temperatures was measured according to the instructions of the kit.

### 2.7 Screening of the concentration of color protection reagents and determination of browning inhibition rat

Each 10 g dry fruiting bodies sample was soaked in 0.5 L reverse osmosis water with 0.4, 0.8, 1.2, and 1.6 g/L of sodium erythorbate; 0.4, 0.8, 1.2, and 1.6 g/L of ascorbic acid; 0.5, 1.0, 1.5, and 2.0 g/L of L‐cysteine; and 2.0, 4.0, 6.0, and 8.0 g/L of citric acid. These samples were rehydrated by soaking at room temperature for five hours. The fruiting bodies were then removed from the water solution for color observation, and 200 μL of the rehydration solution was used to measure the absorbance at the wavelength of 450 nm with a Microplate Absorbance Reader (Hu et al., 2018). The browning inhibition rate was calculated using the following formula.

(5) $$Browning\;\text{inhibition rate}\text{\textbackslash{}\%}\;=\frac{\text{Ao}\;‐\;\text{Am}}{\text{Ao}\;‐\;\text{Ac}}\times 100$$

Ao represents the absorbance of the Yu Muer rehydration water without any color protection reagent. Am represents the absorbance of the Yu Muer rehydration water with different color protection reagents. Ac represents the absorbance of blank water.

### 2.8 Data Analysis

Each experiment was performed in triplicate, and the results were processed using SPSS 23. One‐way ANOVA was performed using LSD, with *p*‐values of <.05 indicating significant level. GraphPad Prism 5.01 was used for graph construction.

## 3 RESULTS

### 3.1 Effect of drying temperature on drying time

**FIGURE 1:**
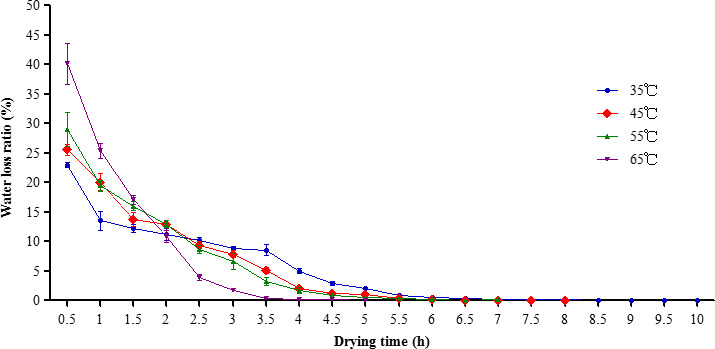
The effect of different drying temperatures on the drying time and water loss ratio

Through the analysis of the effect of the four drying temperatures on the drying time and water loss ratio, we found that for all the drying temperatures, the fruiting bodies lost the majority of their water in the first 3 hr, and the water loss ratio decreased with the extension of drying time. This pattern was similar to the water loss reported in *A. bisporus* during HD (Wang, Liu, & Xu, 2017). Also, we found that for all the drying temperatures, the water loss ratio was the highest at the first 0.5 hr, the order of which was: 65℃ (40.15%)> 55℃ (28.97%)> 45℃ (25.57%)> 35℃ (22.96%). The water loss ratio at 65°C was significantly higher than that of the other temperatures (Figure 1, Table S1). Meanwhile, we measured the surface temperature of the fruiting bodies. At 0.5 hr, the surface temperature of the fruiting bodies reached 35℃ under 65℃ HD, while for 35℃ HD, the surface temperature of the fruiting bodies was 23℃. As the water evaporated rapidly from the fruiting bodies, the increase in the surface temperature of the fruiting bodies accelerated. At 3 hr, the temperature of the fruiting bodies had reached the set drying temperature.

The statistics indicated that, for different drying temperatures, the time required for completing the drying process was: 35℃ (10 hr); 45℃ (8 hr), 55℃ (7 hr), and 65℃ (5 hr). Drying was completed at all of the HD temperatures within 12 hr, which is significantly shorter than the drying time needed for outdoor SD. Drying at 65℃ took 5 hr, which was only half of the time required by drying at 35 ℃. This method not only realizes the completion of drying and packaging on the same day, but also efficiently utilizes the drying equipment, increases the working efficiency, and reduces labor costs.

### 3.2 Effects of different rehydration methods on the color and PPO activity of the fruiting bodies

The dry fruiting bodies subjected to 65℃ HD for 5 hr were used to compare the different rehydration methods. During the room temperature, water rehydration process, the fruiting bodies exhibited browning, becoming a reddish brown to black color. Previous studies have shown that the PPO causes this browning in the fruiting bodies. We determined the PPO activity of the fruiting bodies that were rehydrated using different methods. Also, to explore whether the oxygen in the air affected the oxidation of the fruiting bodies, we compared the difference between film‐wrapped and unwrapped fruiting bodies.

We found that wrapping did not prevent the oxidation reaction of the fruiting bodies, but was able to reduce browning to some extent. Among the three rehydration methods, boiling for 10 min and then soaking for 5 hr resulted in the best fruiting body color. Soaking in boiling water did not avoid browning. The fruiting bodies soaked in water at room temperature were dark reddish, brown, or black. The PPO activity in these fruiting bodies was the highest and was significantly higher than that of the other treatments regardless of whether they were wrapped or not. The order of PPO activity was: soaking at room temperature water >boiling >boiling and then soaking (Table 1). For all three rehydration methods, the PPO activity of the wrapped fruiting bodies was significantly lower than that of the unwrapped fruiting bodies, indicating that the PPO activity could be inhibited to some extent by isolating the fruiting bodies from air.

**TABLE 1:** The effect of different rehydration methods on the color of fruiting bodies and the rehydration water and the PPO activity.

Rehydration methods	Wrap	Water color	Fruiting bodies color	PPO activity（U/g）
Cold water	Unwrapped	Reddish brown	Dark reddish brown	87.23 ± 2.58^a^
	Film‐wrapped	Reddish brown	Dark reddish brown	71.89 ± 1.75^b^
Boiling water	Unwrapped	Light reddish brown	Light reddish brown	42.29 ± 1.76^c^
	Film‐wrapped	Light reddish brown	Light reddish brown	37.62 ± 1.46^d^
Boiled	Unwrapped	Light yellow	White	39.87 ± 1.35^cd^
	Film‐wrapped	Light yellow	White	36.68 ± 1.67^d^

Note:

Different letters in a column indicated significant difference between samples (*p* < .05), while there was no significant difference if a common letter was contained between samples (*p* > .05).

Abbreviation: PPO, polyphenol oxidase.

### 3.3 Effect of Different Drying Temperatures on the Color, FW:DW ratio, and Rehydration Ratio of the Fruiting bodies

**FIGURE 2:**
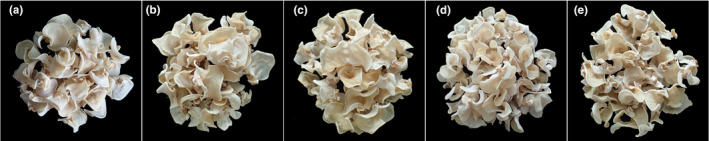
Shape of dried fruiting bodies in different drying temperatures. SD (a); 35℃ HD (b); 45℃ HD (c); 55℃ HD (d); 65℃ HD (e). HD, Hot‐air drying; SD: Sun drying

The color change of edible fungi during drying is an important index for selecting a drying method (Wang et al., 2017). The Yu Muer fruiting bodies have a milky‐white rough surface and a light yellow ventral surface. The color of the dry fruiting bodies and the rehydrated fruiting bodies has a significant influence on the selling price. Therefore, it is particularly important to select a suitable drying temperature. Table 2 shows that as the drying temperature increased, the L* (lightness and darkness), a* (red and green), and b* (yellow and blue) values of the dry fruiting bodies showed irregular changes, and the dry fruiting bodies obtained by HD at four different temperatures showed significant differences in chromaticity. The △E value of the fruiting bodies dried at 35 and 45 ℃ was significantly lower than that of the other treatments, and the color was the closest to that of SD, though the drying took longer. For the fruiting bodies dried at 65℃, the L* value was the highest, representing the highest brightness, and the a* value was the lowest, indicating a small redness, which suggests that the fruiting bodies had good color (Figure 2). The high temperature might inhibit the enzymatic reaction and slow down the enzymatic browning of the fruiting bodies, leading to a light fruiting body color (Xu et al., 2019).

**TABLE 2:** Effects of different drying temperatures on chroma of dried fruiting bodies and rehydration fruiting bodies

Treatments	Dried fruiting bodies						Rehydration fruiting bodies			
	L*	a*	b*	△E	FW:DW ratio	Rehydration ratio	L*	a*	b*	△E
*SD*	75.54 ± 0.14^e^	2.21 ± 0.05^e^	19.30 ± 0.04^c^	–	11.15 ± 0.63^a^	10.77 ± 1.68^a^	61.97 ± 0.73^a^	−1.81 ± 0.01^bc^	6.67 ± 0.08^c^	1.85 ± 0.39^a^
35 ℃HD	75.68 ± 0.02^de^	2.50 ± 0.03^a^	19.48 ± 0.04^b^	0.34 ± 0.11^a^	11.30 ± 0.83^a^	10.92 ± 1.64^a^	62.25 ± 0.44^a^	−1.85 ± 0.03^c^	6.72 ± 0.22^c^	1.75 ± 0.19^a^
45 ℃HD	76.33 ± 0.04^b^	2.35 ± 0.03^b^	19.64 ± 0.11^a^	0.82 ± 0.15^ab^	10.95 ± 0.15^a^	10.83 ± 1.70^a^	61.94 ± 0.78^a^	−1.71 ± 0.03^a^	7.86 ± 0.22^a^	2.95 ± 0.19^b^
55 ℃HD	75.85 ± 0.08^c^	1.71 ± 0.02^c^	18.06 ± 0.10^e^	1.16 ± 0.10^b^	11.02 ± 0.68^a^	10.43 ± 1.92^a^	62.33 ± 1.45^a^	−1.72 ± 0.05^a^	7.10 ± 0.37^b^	2.24 ± 0.82^ab^
65 ℃HD	76.85 ± 0.15^a^	1.97 ± 0.05^d^	19.00 ± 0.08^d^	1.42 ± 0.07^c^	11.01 ± 0.87^a^	10.80 ± 2.44^a^	61.91 ± 1.02^a^	−1.77 ± 0.03^b^	5.91 ± 0.29^d^	1.46 ± 0.82^a^
Fresh	–	–	–	–	–	–	62.99 ± 0.09^a^	−1.82 ± 0.01^c^	5.20 ± 0.17^e^	–

Note:

Different letters in a column indicated significant difference between samples (*p* < .05), while there was no significant difference if a common letter was contained between samples (*p* > .05).

Abbreviations: HD, Hot‐air drying; SD, Sun drying.

The FW:DW ratio of the Yu Muer fruiting bodies at different drying temperatures was recorded and analyzed, and the rehydration ratio was calculated and compared following rehydration. As shown in Table 2 , the FW:DW ratio order was: 55℃ > 35℃ > 65℃ > 45℃, whereas the rehydration ratio order was: 35℃ > 55℃ > 45℃ > 65℃. The fruiting bodies dried at 65 ℃ showed no significant difference in FW:DW ratio and rehydration ratio in comparison with the other treatments, indicating that drying at different temperatures had no significant effect on the weight of the dry fruiting bodies and the rehydrated fruiting bodies.

The fruiting bodies subjected to HD at four different temperatures were rehydrated in boiling water for 10 min and then soaked for 5 hr. The rehydrated fruiting bodies were used for color determination. No significant difference was found in the L* value between the fresh fruiting bodies and rehydrated fruiting bodies previously subjected to different drying treatments. The a* value of the rehydrated fruiting bodies subjected to 45℃ and 55℃ HD was significantly higher than that of the other treatments, and the green color of the rehydrated fruiting bodies subjected to 35℃ HD was closer to that of the fresh fruiting bodies. The b* values of the rehydrated fruiting bodies previously subjected to SD and 35℃ HD differed significantly from that of the rehydrated fruiting bodies subjected to the other treatments, and the difference in b* value between the fresh fruiting bodies and the rehydrated fruiting bodies treated with 65℃ HD was the lowest. Overall, there was no difference in L* and a* values between the rehydrated fruiting bodies previously treated at 65℃ HD and SD.

Additionally, in comparison with the rehydrated fruiting bodies previously subjected to SD, the rehydrated fruiting bodies that were treated at 65 ℃ HD had a significantly lower b* value (lighter yellow) and the lowest △E, indicating that the color of the latter was similar to that of the fresh fruiting bodies, which meets market requirements (Table 2). Drying is a complex process. We only screened the drying temperatures in this study. Predrying and postdrying methods should thus be analyzed in future studies (Lewicki, 2006).

### 3.4 Effect of different drying methods on the main nutrients of the dry products

**FIGURE 3:**
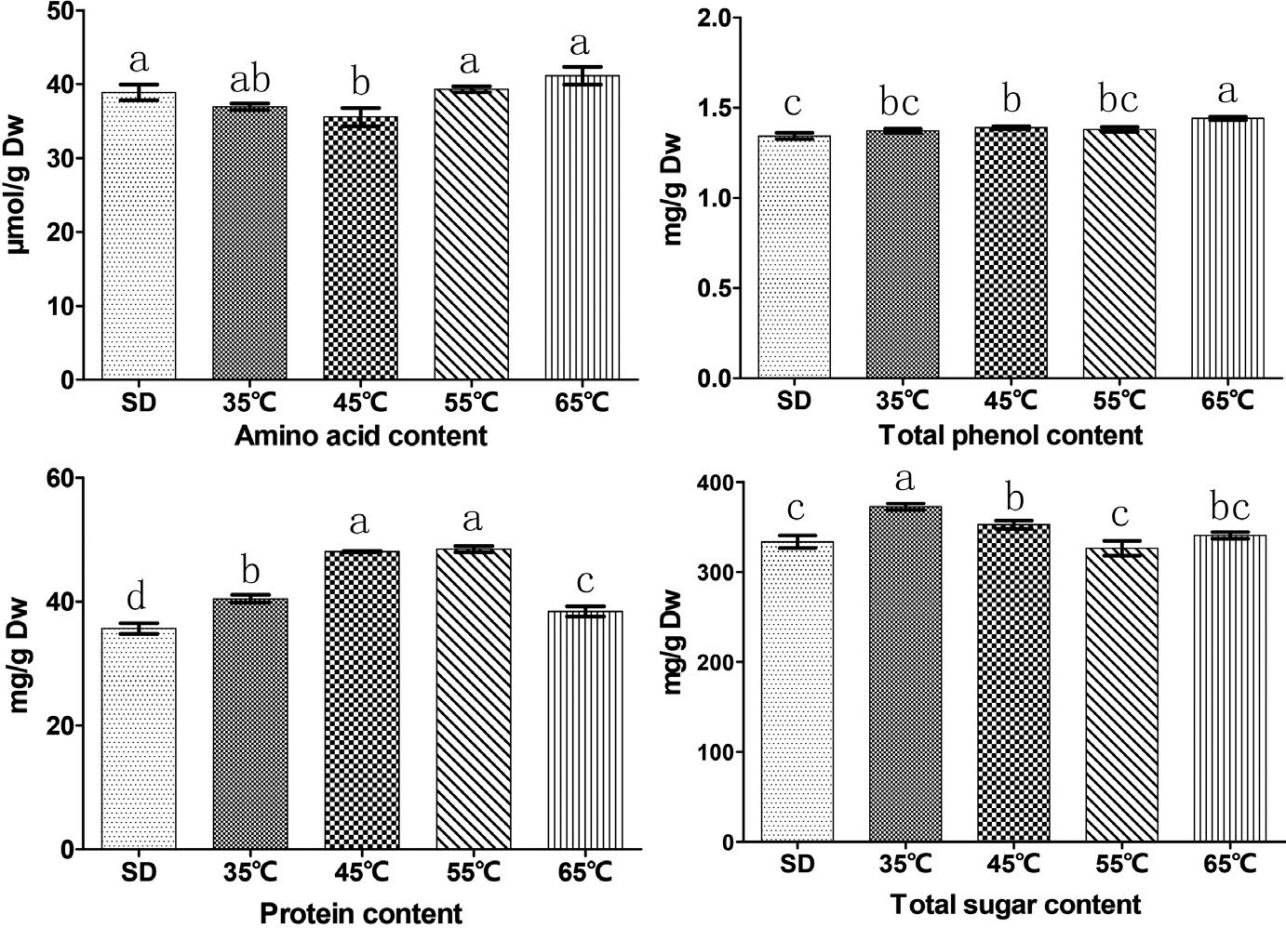
Effects of different drying methods on the contents of amino acid, total phenol, protein, and total sugar of dried fruiting bodies. The same letters are not significantly different (*p* > .05)

Through the analysis of the main nutrients of the dry products processed by different drying methods, we found that the amino acid content of the fruiting bodies dried at different temperatures showed a trend of decreasing first and then increasing as the drying temperature increased, with the highest amino acid content found in the fruiting bodies dried at 65℃ and the lowest seen in the fruiting bodies dried at 45℃ (Figure 3). The content of total phenolics of the fruiting bodies dried at 65 ℃ was the highest and was significantly higher than that of the other treatments. Also, the total phenolic content of the fruiting bodies treated with HD was higher than that of those dried under the sun (Figure 3). The protein content of the fruiting bodies dried at different temperatures showed an overall trend of increasing first and then decreasing as the drying temperature increased, with the protein content of the fruiting bodies dried at 55 ℃ and 45 ℃ being significantly higher than that of the other treatments (Figure 3). This phenomenon might be due to the protein loss caused by the enzymatic reaction and the Maillard reaction at high temperature. The order of total sugar content of the fruiting bodies dried using different treatments was: 35℃>45℃>65℃>SD > 55℃. The total sugar content of the fruiting bodies dried at 35 ℃ was significantly higher than that of the fruiting bodies dried by the other treatments (Figure 3), which may be explained by the degradation of some of the sugar caused by enzymatic reactions at high temperatures . The content of total phenolics and protein of the fruiting bodies dried at 65 ℃ was significantly higher than that of the fruiting bodies subjected to SD. The content of amino acid and total phenolics of the fruiting bodies dried at 65℃ was slightly higher than that of the fruiting bodies dried under the sun. It is evident that drying at 65℃ has little effect on the main nutrients of the fruiting bodies and can thus be used for dry processing.

### 3.5 Effect of color protection reagent on water rehydration at room temperature

**FIGURE 4:**
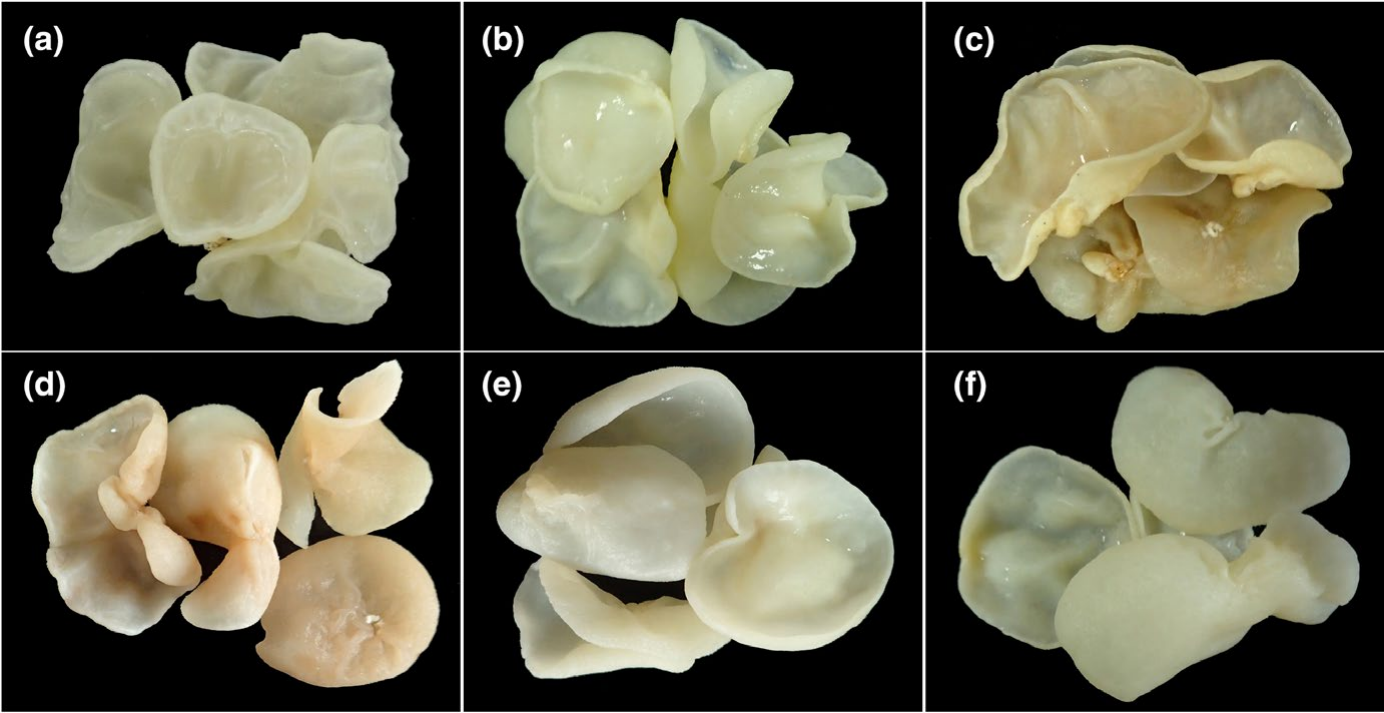
Effects on fruiting bodies’ color with different rehydration methods. fresh fruiting body (a); boiling water rehydration (b); room temperature water rehydration (c); the fruiting bodies turned red following removal from the rehydration water solution consisting of low‐concentration color protection reagents (d); the fruiting bodies turned light red after being removed from the rehydration solution containing low‐concentration color protection reagents (e); the fruiting bodies showed no color change after being removed from the rehydration water solution containing appropriate concentrations of color protection reagents (f)

After being rehydrated in the solutions of four‐color protection reagents of different concentrations, the fruiting bodies were taken out and drained. We found that the fruiting bodies treated with 2 g/L citric acid exhibited a light red color, while the fruiting bodies of all other treatments showed no browning. After draining for 30 min, the fruiting bodies treated with 0.4 and 0.8 g/L sodium erythorbate and ascorbic acid, and 0.5 and 1.0 g/L L‐cysteine and 4 g/L citric acid turned light red, whereas the fruiting bodies treated with 2 g/L citric acid turned dark red. After being removed from the low‐concentration color protection reagents, the fruiting bodies were exposed to the air and became a reddish brown color (Figure 4). Following rehydration, the browning of the water with color protection reagents was less than that of the water without the reagents.

**FIGURE 5:**
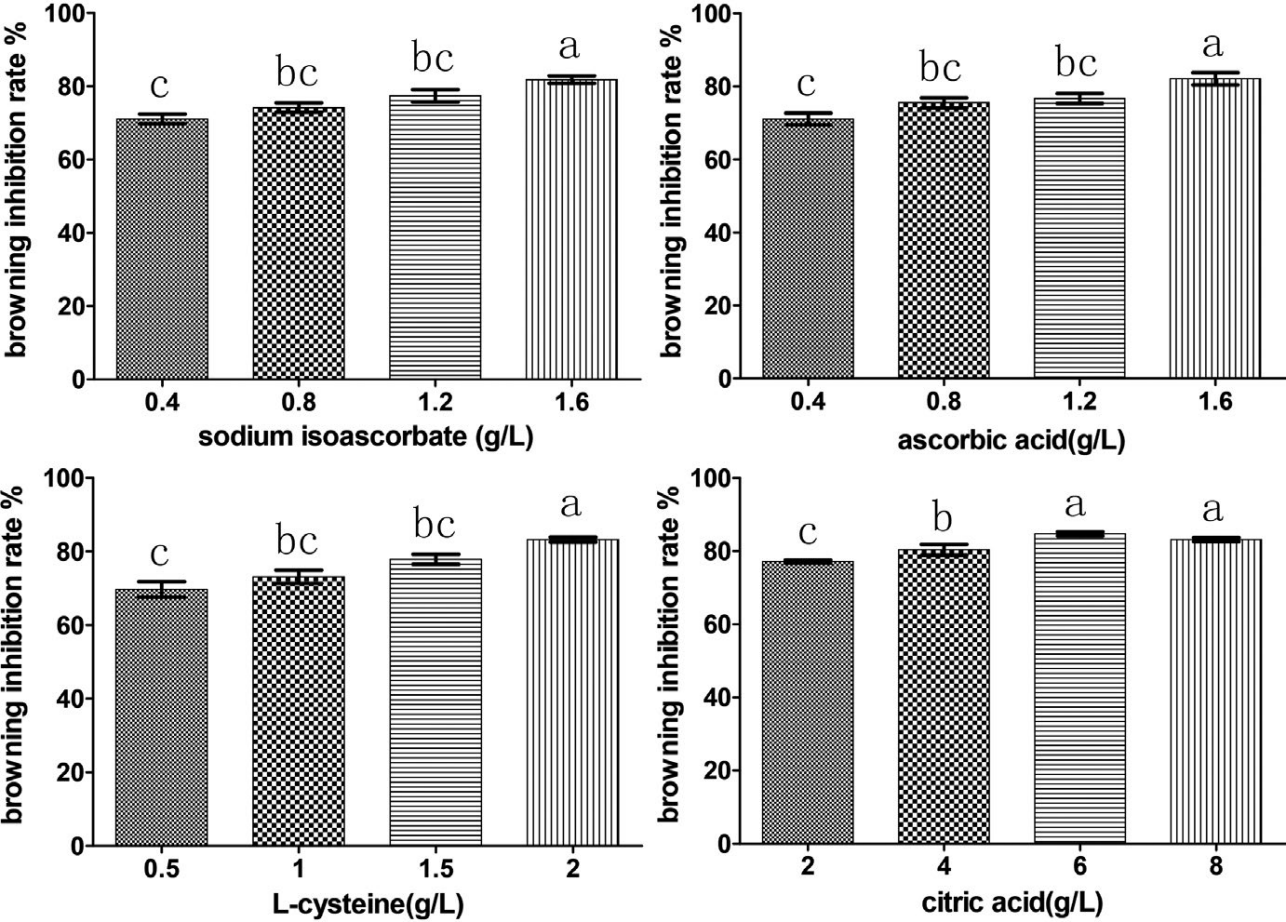
Effects on browning inhibition rate with different concentration of sodium erythorbate, ascorbic acid, L‐cysteine, and citric acid. The same letters are not significantly different (*p* > .05)

The analysis of the browning inhibition rate of the different treatments showed that the treatments with high concentrations of the four‐color protection reagents significantly increased the browning inhibition rate. The browning inhibition rate of sodium erythorbate, ascorbic acid, and L‐cysteine increased as their concentration increased. However, there was no significant difference between the 6 and 8 g/L citric acid treatments, and the browning inhibition rate of the 6 g/L citric acid treatment was higher than that of the 8 g/L treatment. Therefore, the appropriate concentrations of the color protection reagents were as follows: sodium erythorbate 1.6 g/L, ascorbic acid 1.6 g/L, L‐cysteine 2 g/L, and citric acid 6 g/L, wherein the browning inhibition rate of 6 g/L citric acid was the highest (Figure 5). We thus recommend using citric acid as a color protection reagent for room temperature water rehydration, which is also a cost‐effective approach.

## 4 DISCUSSION

Most fresh edible fungi need to be dried in preparation for long‐term storage and marketing, and hot‐air drying (HD) is a typical drying method that is widely used for processing cultivated edible fungi (Fernandes et al., 2013). In this study, we found the water loss ratio at 65°C was significantly higher than that of the other temperatures (Table S1), and need a shortest time for drying. But the drying time can be easily affected by the temperature, water content, and thickness of the ear and the structure and ventilation of the dryer may also affect the drying process (Argyropoulos, Heindl, & Joachim, 2011). Fleshy edible fungi such as *Pleurotus eryngii* and *Lentinus edodes* may not change much in size during the drying process, while the colloidal layer of fresh Yu Muer fruiting bodies has high water content and small fruiting bodies size (Xu et al., 2019), and thus, the holes of the drying tray should not be too large. Suitable hole size in the drying tray would prevent the dry fruiting bodies from falling through during collection, while holes that are too small may obstruct ventilation and prolong the drying time.

Polyphenol oxidase is the key enzyme may lead to the browning or even rotting of fresh edible fungi (Colak et al., 2007; Ding et al., 2016; Ye et al., 2012), thus downgrading their quality. We found the Yu muer fruiting bodies that were boiled and then soaked did not change in color (Table 1), which might be because the high temperature of the boiling water inactivated the PPO (Ludikhuyze, Loey, Smout, & Hendrickx, 2003). This result is consistent with a previous report that the higher the treatment temperature, the lower the activity of mushroom PPO (Baltacıoğlu, Bayındırlı, Severcan, & Severcan, 2015). We observed that the fruiting bodies stretched during boiling, and the water turned pale yellow, and thus, we speculate that some substances might have been released, an observation that requires further exploration.

During the rehydration, the PPO in the fruiting bodies caused browning of both the fruiting bodies and the water. We found that high‐temperature treatment and isolation of the fruiting bodies from the air could inhibit the activity of PPO, thereby reducing the browning effect during rehydration. The fact that the wrapped fruiting bodies still underwent a browning reaction led us to speculate that the oxygen in the water had reacted with the fruiting bodies. All edible fungi contain PPO. The browning of the fruiting bodies by PPO was quite distinct in the Yu Muer fruiting bodies, as the fruiting bodies are white when fresh, demonstrating that browning damages the color of the fruiting bodies. Besides the high‐temperature treatment, processing approaches that work under nonhot conditions, such as ultrasound (US), essential oils, and high‐pressure carbon dioxide (HPCD), could also be adapted to inhibit the PPO reaction (Alikhani‐Koupaei, Mazlumzadeh, Sharifani, & Adibian, 2014; Iqbal et al., 2019; Nasiri, Barzegar, Sahari, & Niakousari, 2019).

Through the analysis of the main nutrients of the dry products processed by different drying methods, we found the highest amino acid content in the fruiting bodies dried at 65℃ and the lowest seen in the fruiting bodies dried at 45℃ (Figure 3). This phenomenon might be because the high temperature promoted protein degradation, which in turn led to an increase in amino acid content (Tian, Zhao, Huang, Zeng, & Zheng, 2015). Phenolic substances are an important physiologically active substance in fungus and can reduce organism damage and disease by regulating the redox state of cells and inhibiting reactive oxygen free radicals (Huang, Cai, & Zhang, 2009). The content of total phenolics of the fruiting bodies dried at 65℃ was the highest and was significantly higher than that of the other treatments. Also, the total phenolic content of the fruiting bodies treated with HD was higher than that of those dried under the sun (Figure 3), which might be due to the nonenzymatic conversion of phenol aldehyde molecules to phenolic substances during the high‐temperature drying process (Que, Mao, Fang, & Wu, 2008).

Yu Muer fruiting bodies were white, and this was most important commodity character. Developing appropriate rehydration methods and screening chemical agents for protecting color during rehydration in the water at room temperature is essential in the development of the Yu Muer industry. In this study, we found the 6 g/L citric acid has the highest browning inhibition rate (Figure 5). We thus recommend using citric acid as a color protection reagent for room temperature water rehydration, which is also a cost‐effective approach. The color protection reagent can be used during the transportation of fresh fruiting bodies, thus extending the shelf life and canning of Yu Muer fruiting bodies. In addition, gamma irradiation, composite color protectors (Fernandes et al., 2012; Ventura‐Aguilar, Colinas‐León, & Bautista‐Baños, 2017), modified atmosphere packaging (MAP) (Ye et al., 2012), covering with active packaging materials, and frozen storage may also be applied in the treatment for keeping the fungus fresh.

In conclusion, through the comparative analysis of HD and SD methods, we found that HD at 65℃ could significantly shorten the drying time of the Yu Muer fruiting bodies. This treatment did not affect the FW:DW ratio and rehydration ratio; the dried and rehydrated fruiting bodies demonstrated better color, and the main nutrients were slightly higher than the fruiting bodies subjected to SD. Soaking after boiling was the most suitable method for rehydration, which could significantly inhibit PPO activity. Four‐color protection reagents were screened, and the suitable concentrations were as follows: 1.6 g/L sodium erythorbate, 1.6 g/L ascorbic acid, 2 g/L L‐cysteine, and 6 g/L citric acid, of which 6 g/L citric acid had the highest browning inhibition rate. Application of high‐temperature HD and color protection reagents during rehydration in the water at room temperature may facilitate the development of the Yu Muer industry.

## CONFLICT OF INTEREST

The authors declare that they have no conflict of interest.

## AUTHORS CONTRIBUTIONS

CTL, BS, and YL acquired funding; YQC conceptualized the research project; LX investigated; YQC, ZWL, FLS, and ZRL performed the experiments; analyzed the data; and drafted the manuscript; YQC and BS curated the data and edited the manuscript.

## ETHICAL APPROVAL

This article does not contain any studies with animals or human participants performed by any of the authors.

## INFORMED CONSENT

Informed consent was obtained from all individual participants included in the study.

## Supporting information

Table S1
