# Supplementary material for: Effect of different drying temperatures on the rehydration of the fruiting bodies of Yu Muer (Auricularia cornea) and screening of browning inhibitors
Source: Food Sci Nutr. 2020 Sep 27;8(11):6037–46. doi: 10.1002/fsn3.1891 (PMC7684618; doi:10.1002/fsn3.1891)
Supplement: Supplementary file 1 — Table S1 [file FSN3-8-6037-s001.docx]

**SUPPLEMENTARY TABLE 1**

The effect of different drying temperatures on the drying time and water loss ratio

| **Drying time (h)** | | **Drying temperature (℃)** | | | |
| --- | --- | --- | --- | --- | --- |
|  | **35 ℃** | | **45 ℃** | **55 ℃** | **65 ℃** |
| 0.5 | 22.96±0.72^b^ | | 25.57±1.67^b^ | 28.97±4.92^b^ | 40.15±5.99^a^ |
| 1 | 13.52±2.72^c^ | | 20.08±2.55^b^ | 19.50±1.59^b^ | 25.38±2.10^a^ |
| 1.5 | 12.19±1.15^c^ | | 13.76±2.05^b^ | 16.03±1.42^ab^ | 17.06±1.24^a^ |
| 2 | 11.22±1.79^a^ | | 12.87±0.68^a^ | 12.97±1.10^a^ | 10.91±1.82^a^ |
| 2.5 | 10.20±0.73^a^ | | 9.41±0.99^a^ | 8.72±1.23^a^ | 3.96±0.86^b^ |
| 3 | 8.87±0.64^a^ | | 7.91±1.04^a^ | 6.59±2.33^a^ | 1.79±0.15^b^ |
| 3.5 | 8.57±1.70^a^ | | 5.17±0.91^b^ | 3.26±1.27^b^ | 0.42±0.10^c^ |
| 4 | 4.96±0.77^a^ | | 2.1±0.40^b^ | 1.70±0.90^b^ | 0.11±0.01^c^ |
| 4.5 | 2.88±0.61^a^ | | 1.22±0.21^b^ | 0.86±0.42^b^ | 0.13±0.09^c^ |
| 5 | 2.09±0.12^a^ | | 1.01±0.8^b^ | 0.58±0.48^bc^ | 0.08±0.03^c^ |
| 5.5 | 0.91±0.24^a^ | | 0.38±0.02^b^ | 0.32±0.27^b^ | - |
| 6 | 0.55±0.14^a^ | | 0.25±0.13^b^ | 0.17±0.15^b^ | - |
| 6.5 | 0.34±0.10^a^ | | 0.08±0.04^b^ | 0.11±0.11^b^ | - |
| 7 | 0.23±0.08^a^ | | 0.06±0.01^a^ | 0.20±0.16^a^ | - |
| 7.5 | 0.15±0.09 | | 0.05±0.01 | - | - |
| 8 | 0.12±0.07 | | 0.08±0.04 | - | - |
| 8.5 | 0.07±0.05 | | - | - | - |
| 9 | 0.04±0.02 | | - | - | - |
| 9.5 | 0.04±0.02 | | - | - | - |
| 10 | 0.05±0.03 | | - | - | - |

*Note*: Different letters in a column indicated significant difference between samples (P < 0.05), while there was no significant difference if a common letter was contained between samples (P > 0.05)
